# Promising non-invasive biomarkers for kidney allograft monitoring: a mini review

**DOI:** 10.3389/fimmu.2026.1801117

**Published:** 2026-06-29

**Authors:** Luke Barr, Fareeha Mubin, John Friedewald, Sook Hyeon Park

**Affiliations:** 1 Department of Medicine, Division of Nephrology and Hypertension, Northwestern University, Feinberg School of Medicine, Chicago, IL, United States; 2 Department of Medicine, Division of Nephrology, Jefferson Einstein Philadelphia Hospital, Philadelphia, PA, United States

**Keywords:** donor-derived cell-free DNA, gene expression profile, immunologic monitoring, kidney transplant, noninvasive biomarkers, urine chemokines

## Abstract

Significant advancements have been made in kidney transplantation, with a wealth of immunosuppressive and antimicrobial therapies that have significantly improved graft survival. Despite these accomplishments, the ability to promptly identify kidney allograft pathology for these therapies has been limited by the reliance on kidney biopsy as the gold-standard diagnostic modality, which is invasive. To overcome these limitations, non-invasive biomarkers have been developed over the past two decades. Donor-derived cell-free DNA, gene expression profiling, urinary chemokines, extracellular vesicles, microRNA, and Torque Teno Virus are candidates near or already in clinical use. This mini-review discusses the strengths and limitations of these biomarkers for the surveillance and prognostication of renal allografts.

## 1 Introduction

Significant advances in immunosuppressants, effective treatments for infectious complications, and surgical techniques have improved overall outcomes in kidney transplantation (1). Despite this progress, immunologic allograft injury remains a key contributor to long-term graft loss, and early detection of rejection remains limited (2). Although kidney transplant biopsies are considered the gold standard for diagnosing rejection, they can be risky and expensive, and are subject to sampling error and significant inter- and intra-observer variability (3, 4).

Over the past decade, numerous non-invasive biomarkers have been proposed for detecting alloimmune activity. Several of these are already being used for routine post-transplant rejection monitoring in the US, but their diagnostic performance varies. This mini-review focuses on emerging non-invasive biomarkers from blood or urine that are near or in clinical use, specifically donor-derived cell-free DNA (dd-cfDNA), Gene Expression Profiling (GEP), urine chemokines, CXCL-9, CXCL-10, exosomes, extracellular vesicles (EV), microRNA, and Torque Teno Virus (TTV). We evaluated the performance and evidence supporting each biomarker, addressed their limitations, and highlighted potential future directions.

## 2 Donor-derived cell-free DNA

Dd-cfDNA has been shown to correlate with graft injury, and levels are high immediately post-transplantation (5). Following transplantation, dd-cfDNA levels rapidly decline within 1 week to <0.5% in stable, quiescent grafts (6). Thus, the presence of a low-level graft damage-specific biomarker makes dd-cfDNA a promising candidate for graft surveillance (6). Dd-cfDNA can be reported as a percentage of dd-cfDNA relative to total cfDNA and as an absolute quantity (7). Dd-cfDNA diagnostic performance characteristics are summarized in **Table 1**.

**Table 1:** Summary of diagnostic characteristics of donor-derived cell-free DNA and gene expression profiling.

Donor-dervied cell-free DNA									
Author	Biomarker	Study design	Biopsy indication	Rejection type	Sens	Spec	PPV	NPV	AUC
Akifova et al. (2024) (14)	Droplet Digital	Open label randomized clinical trial single center	Abnormal dd-cfDNA or for cause	ABMR			0.77	0.85	
Loupy et al. (2024) (16)	Allosure and AlloSeq	Prospective multicenter	Surveillance and for cause	Acute Rejection	–	–	–	–	0.76
Aubert et al. (2024) (10)	Allosure and AlloSeq	Prospective observational multicenter	Surveillance and for cause	Acute Rejection	–	–	–	–	0.82
				ABMR	–	–	–	–	0.84
				TCMR	–	–	–	–	0.78
Halloran et al. (2022) (13)	Prospera	Prospective observational multicenter (Trifecta)	For cause	ABMR (% dd-cfDNA)	–	–	–	–	0.86
				ABMR (quantity dd-cfDNA)	–	–	–	–	0.85
Bu et al. (2022) (11)	Allosure	Prospective observational multicenter (Admiral)	Surveillance and for cause	Acute Rejection	0.78	0.71	0.50	0.90	0.80
				ABMR	0.79	0.59	0.42	0.88	0.80
				TCMR	0.75	0.50	0.36	0.84	0.70
Halloran et al. (2022) (9)	Prospera	Prospective observational multicenter (Trifecta)	For cause	Acute Rejection (Histology)	0.74	0.81	0.71	0.83	0.82
				Acute Rejection (MMDx)	0.83	0.81	0.68	0.91	0.88
Park et al. (2021) (18)	TRAC	Prospective observational (*post hoc* analysis) multicenter	Surveillance	SubAR	0.47	0.88	0.56	0.84	0.72
Huang et al. (2019) (15)	Allosure	Prospective observational single center	For cause	Acute Rejection	0.79	0.72	0.77	0.75	0.71
				ABMR	1.00	0.72	0.69	1.00	0.82
				TCMR	–	–	–	–	0.42
Jordan et al. (2018) (12)	Allosure + DSA	Subgroup analysis of prospective observational multicenter (DART)	For cause	ABMR	0.81	0.82	0.81	0.83	0.86
Sigdel et al. (2018) (17)	Prospera	Retrospective analysis single center	For cause and surveillance	Acute Rejection	0.89	0.73	0.52	0.95	0.87
				SubAR	0.92	0.75	0.55	0.97	0.89
Bloom et al. (2017) (8)	Allosure	Prospective Observational Multicenter (DART)	For cause	Acute Rejection	0.59	0.85	0.61	0.84	0.74
				ABMR	0.81	0.83	0.44	0.96	0.87
Gene Expression Profiling									
Author	Biomarker	Study Design	Biopsy Indication	Rejection Type	Sens	Spec	PPV	NPV	AUC
Bestard et al. (2024) (24)	Tutivia	Multicenter prospective	Surveillance For-cause	Acute rejection	–	–	0.60	0.79	0.69
Friedewald et al. (2019) (21)	TruGraf	Prospective, multicenter	Surveillance	ABMR TCMR Any rejection	0.38 0.48 0.47-66	0.85 0.85 0.8-87	0.33 0.37 0.47-0.61	0.87 0.9 0.78-0.88	0.71 0.80 0.85
Zhang et al. (2019) (25)	Targeted 17-gene assay (TREx)	Multicenter retrospective [GoCar cohort]	Surveillance	Acute rejection	–	–	0.98	0.79	0.83
Roedder et al. (2014) (28)	kSORT (13, 12-gene models)	Multicenter prospective, internal training and validation sets	Surveillance and for-cause	Acute rejection	Training set: 83% Validation set: 91%	Training set: 91% Validation set: 99%	Training set: 92% Validation set: 98%	Training set: 81% Validation set: 95%	Training set: 0.94 Validation set: 0.95
Van Loon et al. (2021) (29)	kSORT (13 gene models)	Retrospective Multicenter	Surveillance and for-cause	Acute rejection	0.17	0.94	0.5	0.76	0.51
Akalin et al (2021) (26)	AlloMAP	Prospective, multicenter cohort with retrospective biomarker development and validation	Surveillance and for-cause	Acute rejection	0.76	0.73	0.42	0.93	0.80

ABMR, antibody-mediated rejection; AUC, area under the curve; dd-cfDNA, donor-derived cell-free DNA; EU-TRAIN, European Transplantation and Innovation; MMDx, Molecular Microscope Diagnostic System; PPV, positive predictive value; NPV negative predictive value; subAR, subclinical rejection; TCMR, T-cell mediated rejection.

### 2.1 DD-cfDNA fraction across rejection phenotypes

The literature exploring dd-cfDNA and rejection phenotypes has conflicting results. While useful for identifying the degree or severity of graft injury, the available data do not support its use to discriminate between mechanisms of immunologic injury. In the Diagnosing Active Rejection in Kidney Transplant Recipients (DART) study of clinical acute rejection, the median dd-cfDNA varied by rejection type, at 2.9% in antibody-mediated rejection (ABMR), 1.2% in T cell-mediated >1B, 0.2% in T cell-mediated 1A, and 0.3% in no active rejection (8). Only T cell-mediated rejection (TCMR) graded 1B or higher was statistically significant relative to controls (p = 0.05) (8). The Trifecta study similarly stratified the dd-cfDNA fraction by rejection phenotype identified on biopsy using the Molecular Microscope Diagnostic System (MMDx) gene expression and conventional histology (9). For rejection identified by MMDx, the median dd-cfDNA was 2.11% for ABMR, 1.61% for TCMR, and 1.56% for mixed rejection, while no rejection was 0.3% (9). While these studies suggest increased expression in ABMR relative to TCMR, Aubert et al. found that fractional dd-cfDNA increased to a greater extent in TCMR (2.03%) than in ABMR (1.15%). This study also showed that dd-cfDNA had an increasing correlation with the severity of Banff lesion grades in both ABMR and TCMR (10). The literature has established that dd-cfDNA is increased with immunologic injury correlating with the degree of active rejection, but does not clearly differentiate rejection phenotypes.

### 2.2 Dd-cfDNA in active rejection

Dd-cfDNA has been studied for discriminating active rejection from healthy allografts. The Admiral study reported that for active rejection, dd-cfDNA had an area under the curve (AUC) of 0.80, positive predictive value (PPV) 0.5, negative predictive value (NPV) 0.9, and outperformed serum creatinine AUC 0.49 (11). In the DART study, dd-cfDNA discriminated clinical acute rejection, with an AUC of 0.74, PPV of 0.61, and NPV of 0.83 (8).

Jordan et al. studied whether dd-cfDNA can complement donor-specific antibody (DSA) in diagnosing ABMR (12). The combination of dd-cfDNA > 1% and positive DSA discriminated ABMR, with an AUC of 0.86, PPV of 0.81, and NPV of 0.83 (12). Halloran et al. reported that dd-cfNDA outperformed DSA to diagnose ABMR; absolute dd-cfDNA quantity (AUC 0.85), dd-cfDNA fraction (AUC 0.86), compared with DSA (AUC 0.66) (13). Akifova et al. reported that dd-cfDNA had a PPV of 0.77 and an NPV of 0.85 for ABMR detection, identifying rejection a median of 2.8 months earlier than controls (14). These results suggest that dd-cfDNA could expedite the identification via allograft biopsy and timely treatment of ABMR.

Dd-cfDNA has not consistently demonstrated the ability to identify TCMR. Huang et al. reported that dd-cfDNA could not differentiate TCMR, with an AUC of 0.42 (15). Conversely, Aubert et al. found an AUC 0.78 for TCMR (10). As previously described, dd-cfDNA correlates immunologic injury with increasing histologic grade in both ABMR and TCMR (16). The inconsistency in these data may reflect variability in patient populations, with differing percentages of high-grade TCMR or borderline changes within rejection cohorts.

### 2.3 Dd-cfDNA in subclinical acute rejection

The utility of dd-cfDNA has also been studied for detecting subclinical acute rejection (subAR). Sigdel et al. reported an AUC of 0.89 for discriminating subAR. PPV was 0.55, and NPV was 0.97 (17). In a *post-hoc* analysis, Park et al. reported an AUC of 0.72, PPV of 0.56, and NPV of 0.84 for subAR (18). For subclinical ABMR alone, the AUC was 0.84 (18). Overall, despite limited data, the high NPV suggests that dd-cfDNA may be useful for excluding subAR, particularly in subclinical ABMR.

### 2.4 Dd-cfDNA as a prognostic marker

Stites et al. assessed clinical outcomes in patients with TCMR 1A/borderline rejection using serial dd-cfDNA measurements (19). They found that elevated dd-cfDNA (≥ 0.5%) was associated with adverse clinical outcomes over 6 months, including decline in eGFR (p=0.004), increased *de novo* DSA 40% vs. 2.7% (p<0.0001), and higher rates of future or persistent rejection 21.4% vs. 0% (p=0.003) (19). Bunnapradist et al. reported that serial dd-cfDNA trends could predict one-year outcomes, including graft loss, subsequent biopsy-proven acute rejection, post-biopsy DSA, and/or lack of resolution of renal dysfunction (20). At one year, those with consistently low (<1%) dd-cfDNA and those with initially high (≥1%) dd-cfDNA that subsequently declined below 1% had a positive outcome 60% of the time (20). These data highlight that even in non-diagnostic scenarios, dd-cfDNA may help with prognostication and the identification of at-risk patients.

In summary, dd-cfDNA is a non-specific marker of allograft damage that correlates with the degree of immunologic injury. It has been used to distinguish active rejection from healthy allografts, but its modest PPV limits its ability to detect active rejection. However, dd-cfDNA has shown promise to augment DSA for the identification of ABMR. And in subAR, high NPV of dd-cfDNA provides a non-invasive means to potentially exclude rejection, as well as prognostic ability to identify at-risk patients who need closer longitudinal monitoring.

## 3 Gene expression profiling

Peripheral blood gene expression profiles (GEPs) are another potential tool to identify or exclude immune-mediated graft injury or immune quiescence (18, 21, 22). GEP diagnostic performance characteristics are summarized in **Table 1**.

### 3.1 TruGraf

TruGraf (Eurofins Transplant Genomics) is one of the commercially available GEP tests. A 57-gene microarray-based GEP was developed in the Clinical Trials in Organ Transplantation (CTOT)-08 study by Friedewald et al. (21). Surveillance biopsies were paired with TruGraf measurements in patients with stable allograft function. The NPV ranged from 78% to 88%, and the PPV ranged from 47% to 61% (21). The high NPV for subAR suggests its potential usefulness as an alternative to surveillance biopsy in stable allografts.

Park et al. evaluated the performance of TruGraf alone and in combination with plasma dd-cfDNA in kidney transplant patients with stable allograft function who had either normal histology or subAR on surveillance biopsy (18). When both were negative, the NPV increased to 88%, and when both were positive, the PPV rose to 81% (18). There is some evidence linking subAR in kidney transplant patients to worse clinical outcomes, although this is not well established yet (21, 23).

### 3.2 TREx and Tutivia

TREX and Tutivia (Verici Dx) are gene-expression-based biomarkers developed based on the Genomics of Chronic Allograft Rejection Study (GoCAR) (24, 25). Zhang et al. developed and validated 17-gene signature assay to diagnose acute cellular rejection at 3 months post-transplant and validated using TREx (sequence-based targeted expression) (25). The TREx assay successfully identified subAR, including borderline changes at 3 months post-transplant, with a PPV of 0.73 and an NPV of 0.89 (25). Bestard et al. developed a 17-gene signature (Tutivia) for acute rejection within 6 months post-transplant, which showed an AUC 0.69, NPV 0.79, and PPV 0.60 (24).

### 3.3 AlloMap

AlloMap Kidney is a 5-gene assay that uses next-generation sequencing to differentiate immune activity, including subAR, from inactivity and was validated in a case-control study (26, 27). The AUC in the primary validation set, differentiating rejection from quiescence, was 0.79; in the second validation set, it was 0.80; however, sample sizes were small (26).

### 3.4 kSORT

The kidney Solid Organ Response Test (kSORT), is a 17-gene PCR-based expression assay that initially showed promising results in identifying acute rejection in a case-control design and correlation-based algorithm (AUC = 0.94) (28). In a recent prospective multicenter cohort study utilizing “real-world” rejection prevalence, the kSORT assay failed to identify rejection (AUC 0.51) reliably (29).

Different GEPs comprise varying gene combinations, many of which have not been validated in large-scale external cohorts and lack direct comparison. Additional studies are needed to validate GEPs as precision diagnostic indicators.

## 4 Urine chemokines

Early research showed that urine CXCL9 and CXCL10 levels were significantly higher (p=0.0002) during episodes of acute rejection and BK infection (p=0.0002) but not elevated in non-inflammatory conditions such as stable graft function, calcineurin toxicity, and interstitial fibrosis with tubular atrophy (30). Urinary chemokine diagnostic performance characteristics are summarized in **Table 2**.

**Table 2:** Summary of diagnostic characteristics of urinary chemokines.

Urine Chemokines									
Author	Biomarker	Study design	Biopsy indication	Rejection type	Sens	Spec	PPV	NPV	AUC
Goutaudier et al. (2025) (41)	CXCL 9 and CXCL 10 using Ella Immunoassay	Prospective cohort multicenter	For cause and surveillance	Acute Rejection (CXCL 9)	0.62	0.72	0.12	0.97	0.70
				Acute Rejection (CXCL 10)	0.67	0.60	0.10	0.97	0.64
Arnau et al. (2021) (34)	CXCL10/Cr	Retrospective analysis, single center	For cause and surveillance	ABMR	0.75	0.71	–	–	0.76
				SubABMR	0.83	0.78	–	–	0.80
				TCMR	0.72	0.68	–	–	0.72
				SubTCMR	–	–	–	–	0.78
Tinel et al. (2020) (38)	Integrative model using CXCL9 and CXCL10	Retrospective cohort multicenter	For cause and surveillance	Acute Rejection	0.84	0.69	0.47	0.93	0.85
				SubAR	–	–	–	–	0.81
Rabant et al. (2016) (33)	CXCL9/Cr and CXCL10/Cr	Prospective cohort single center	For cause and surveillance	Acute Rejection (CXCL9/Cr)	–	–	–	–	0.72
				Acute Rejection (CXCL10/Cr)	–	–	–	–	0.74
				SubAR (CXCL9/Cr)	–	–	–	–	0.57
				SubAR (CXCL10/Cr)	–	–	–	–	0.64
Rabant et al. (2015) (32)	CXCL9 and CXCL10	Retrospective cohort single center	For cause	Acute Rejection (CXCL 9)	0.58	0.85	0.59	0.84	0.72
				Acute Rejection (CXCL 10)	0.59	0.83	0.58	0.84	0.74
Hricik et al. (2013) (31)	CXCL9 and CXCL10	Prospective observational multicenter	For cause and surveillance	Acute Rejection (CXCL9)	0.85	0.81	0.68	0.92	0.86
				Acute Rejection (CXCL10)	0.74	0.86	0.71	0.88	0.77

### 4.1 Urine chemokines in active rejection

In the CTOT-01, urine CXCL9 (AUC 0.86, PPV 0.68, NPV 0.92) and CXCL10 (AUC 0.77, PPV 0.71, NPV 0.88) performed well for active rejection, with particularly high NPV (31). In contrast, Rabant et al. did not observe similar performance with AUCs of 0.72 and 0.74 for CXCL9 and CXCL10, respectively, for active rejection (32). While both studies included patients with BK virus, CTOT-01 included only one patient with BK infection, while the second study included 12 (31, 32). This considerable confounder may have contributed to the performance discrepancy.

### 4.2 Urine chemokines in subclinical rejection

Rabant et al. reported poor discrimination of subAR, with CXCL9:Cr AUC of 0.57 and CXCL10:Cr AUC of 0.64 (33). This study used an unselected population, including BK infection, which provides generalizability but may limit the study’s performance. Arnau et al. reported that CXCL10/Cr had an AUC of 0.80 for subclinical ABMR and 0.78 for subclinical TCMR (34). Other studies have also shown strong performance, particularly for CXCL10 and subclinical TCMR (35).

A randomized controlled trial assessed the performance of serial CXCL10-triggered biopsies compared with standard of care alone. Although no improvement in outcomes was observed, CXCL10-triggered biopsies detected rejection more than 1-year surveillance biopsies (20% vs. 10%, respectively; P = 0.02), suggesting potential for monitoring subAR (36).

### 4.3 Effort to enhance chemokine performance

The significant heterogeneity in urine chemokines studies has partly been due to confounding factors, such as urinary tract infections and BK virus, which play a role (37). Tinel et al. developed an integrative model to enhance its applicability to clinical practice. Their multiparametric model, which included clinical factors, DSA, CXCL9, and CXCL10, effectively distinguished clinical rejection with AUC 0.85, and subAR with AUC 0.81 (38).

Another limitation to reliably studying chemokines is the lack of reproducible assays for broad-scale clinical practice (39). Tinel et al. validated the Ella (ProteinSimpleTM) system for analyzing CXCL9 and CXCL10, showing good intra- and inter-assay precision and completing analysis in 90 minutes vs. 7 hours for standard ELISA, which could provide faster results to aid clinical decision-making (40). While incorporating an integrative model improved CXCL9 performance AUC 0.78, it had no meaningful impact on CXCL10 (41).

Urine chemokines remain a promising biomarker for risk stratification between rejection and non-rejection, but rejection should be confirmed by biopsy. Advances in addressing clinical confounders, particularly the use of integrative models, demonstrate the potential to incorporate chemokines into allograft monitoring. Additionally, progress in standardizing chemokine measurements will facilitate widespread, reliable adoption.

## 5 Extracellular vesicles

Extracellular vesicles (EV) are lipid bilayer vesicles released by all cells that contain DNA, mRNA, microRNA, cytosolic and cell surface proteins, lipids, and other metabolites, including inflammatory cytokines (42). EV diagnostic performance characteristics are summarized in **Supplementary Table 1**.

### 5.1 Extracellular vesicles in acute rejection

Sigdel et al. examined urine exosomes and identified 11 proteins that were significantly different between the rejection and no-rejection groups (p < 0.05) (43). Fekih et al. studied mRNA in urinary exosomes and identified a multigene signature with strong diagnostic performance for rejection, with an AUC of 0.93, NPV of 0.93, and PPV of 0.86 (44). To improve widespread implementation, Fekih et al. published a recent paper developing Exosome Transplant Rejection Urine (ExoTRU), a custom exosomal assay using real-time PCR available in clinical laboratories (45). They identified a 4-gene signature that achieved an AUC of 0.73, outperforming serum creatinine’s AUC of 0.51 (p=0.00048) (45). Lim et al. compared patients with biopsy-proven TCMR to those with stable graft function (SGF) and identified two overexpressed proteins that together distinguished TCMR from SGF, with an AUC of 0.74 (46).

EVs and specifically urine exosomes, provide a wealth of potential signatures across various clinical phenotypes. However, research on EVs is limited to small sample sizes, and the capacity of EVs to monitor allograft status remains unknown. Additionally, due to the multitude of targets, existing studies lack reproducibility. Finally, while ExoTRU shows promise, standardizing isolation techniques is necessary to enable widespread use and improve external validity.

## 6 MicroRNA

An individual microRNA can target multiple mRNAs, and a single mRNA can be targeted by numerous microRNAs (47), which can serve as molecular signatures of clinical conditions such as graft rejection. MicroRNA diagnostic performance characteristics are summarized in **Supplementary Table 1**.

Seo et al. identified a three-microRNA signature that distinguished acute rejection with AUC of 0.85 and 0.77, discovery and validation cohorts, respectively (48). Using peripheral blood cells, Matz et al. reported a five-microRNA signature AUC 0.97 for vascular TCMR versus stable graft function (49). Similarly, the same author isolated microRNAs from plasma to distinguish vascular TCMR but found only three differing microRNAs, with lower diagnostic performance, highlighting variability across mediums and challenges with reproducibility (50).

MicroRNAs have shown potential for graft monitoring; however, challenges remain. While they are easily isolated from various sources with variable expression across different medums (49–51). Most studies of MicroMRNA were small case-control designs, which contribute to heterogeneity of results and limit their generalizability.

## 7 Torque teno virus

TTV is considered a near-ubiquitous, apathogenic component of the human virome (52) and has emerged as a promising marker for monitoring net immunity of kidney transplant recipients (53–55). However, the lack of standardized thresholds and the absence of interventional trial results currently limit clinical adoption. At present, TTV is more accurately characterized as a promising risk-stratification biomarker for monitoring immunosuppression intensity than as a diagnostic test for rejection. TTV diagnostic performance characteristics are summarized in **Supplementary Table 1**.

## 8 Future directions

Currently, dd-cfDNA and GEP have received coverage approval from the Centers for Medicare and Medicaid Services (CMS) and are being adopted into clinical practice in the US. Furthermore, the European Society of Organ Transplantation (ESOT) consensus statement has endorsed the use of non-invasive biomarkers, specifically dd-cfDNA, GEP, and urine chemokines, in specific clinical situations (56).

While the strong NPV of biomarkers in use is being used to rule out rejection, the more limited PPV must be interpreted with caution to avoid unnecessary for-cause biopsies. The gold standard for rejection diagnosis—kidney biopsy and histologic interpretation—is imperfect. While the Banff criteria aim to standardize the field, their interpretation relies on pathologists’ training and experience, and sampling errors (57). Most noninvasive biomarkers were developed based on histologic interpretation. Two potential methods have been developed to address this limitation. The Molecular Microscope Diagnostic System (MMDx) uses microarrays to assess mRNA expression in biopsy samples (58). The Banff Human Organ Transplant Panel (B-HOT) is an alternative to whole-transcriptome-based approaches, such as MMDx (59). Continued refinement of our gold-standard histology, alongside MMDx and B-HOT, will hopefully improve the ability to diagnose rejection more accurately and the quality of future non-invasive biomarker development and validation.

Additional common limitations in biomarker research include small sample sizes and a lack of standardization in analytical techniques, sample processing, and histology interpretation. To address these issues, the European BIOMARGIN program has been developed to identify biomarker candidates first through case-control studies, then to evaluate their diagnostic performance using extensive data and samples (60). Through this comprehensive approach, the group hopes to develop biomarkers with high levels of evidence (60).

While non-invasive biomarkers are emerging tools for graft surveillance, they do not exist in isolation. In the study design, potential biomarkers are incorporated with available clinical data, including allograft biopsy, serum creatinine, other clinical data, and HLA antibody status and matching. A real-world approach is to use these data in concert. Aubert et al. reported that adding dd-cfDNA to a standard clinical care model could increase the performance of dd-DNA on rejection detection (10). Similarly, Tinel et al. suggested that combining clinical data with urine chemokines (CXCL-9 and CXCL-10) could improve the biomarkers’ performance (38). EU-TRAIN is a European consortium with this end in mind (61). Their goal is to use multi-dimensional data to develop a prediction system called EU-TRACER (61). This will allow for early risk stratification to promote early intervention, such as biopsy and prognostication of allograft survival (61). This will provide a standardized approach to integrating non-invasive biomarkers with other clinical data to optimize the standard of care (61).

Currently, there is not enough evidence to fully replace biopsies with diagnoses based on non-invasive biomarkers. Additional validation, preferably through prospective randomized clinical trials, is necessary. Despite this, biomarkers are being used in practice to support graft surveillance and reduce the need for biopsies. Additionally, the many existing candidates show great promise in contributing to a multimodal, patient-centered approach that may improve patient outcomes.
