# Supplementary material for: Promising non-invasive biomarkers for kidney allograft monitoring: a mini review
Source: Front Immunol. 2026 Jun 29;17:1801117. doi: 10.3389/fimmu.2026.1801117 (PMC13357181; doi:10.3389/fimmu.2026.1801117)
Supplement: Supplementary file 1 [file Table1.docx]

Supplementary Table 1: Summary of Diagnostic Characteristics of Extracellular Vesicles, microRNA and Torque Teno Virus

| **Extracellular Vesicles** | | | | | | | | | |
| --- | --- | --- | --- | --- | --- | --- | --- | --- | --- |
| Author | Biomarker | Study Design | Biopsy Indication | Rejection Type | Sens | Spec | PPV | NPV | AUC |
| El Fekih et al (2025)^1^ | Urine exosome (mRNA multigene signature) | Prospective observational multicenter | For cause | Acute Rejection | 0.93 | 0.43 | 0.45 | 0.93 | 0.73 |
| El Fekih et al (2021)^2^ | Urine exosome (mRNA multigene signatures) | Prospective observational  Multicenter | For cause | Acute Rejection | 0.85 | 0.94 | 0.86 | 0.93 | 0.93 |
| Lim et al (2018)^3^ | Urine exosome (tetraspanin-1 and hemopexin) | Cross-sectional case control multicenter | For cause | TCMR | 0.64 | 0.73 | - | - | 0.74 |
| **microRNA** | | | | | | | | | |
| Author | Biomarker | Study Design | Biopsy Indication | Rejection Type | Sens | Spec | NPV | PPV | AUC |
| Seo et al (2023)^4^ | Three microRNA signature | Cross sectional multicenter | For cause and surveillance (n=108 discovery, n=260 validation) | Acute Rejection (discovery) | - | - | - | - | 0.85 |
|  |  |  |  | Acute Rejection (validation) | 0.99 | 0.26 | 0.63 | 0.95 | 0.77 |
| Matz et al (2016)^5^ | Five microRNA Signature from blood | Case control single center | For cause | Vascular TCMR | 1.00 | 0.90 | - | - | 0.97 |
| Matz et al (2016)^6^ | Three microRNA Signature from plasma | Case control single center | For cause | Vascular TCMR | - | - | - | - | 0.63 |
| **Torque Teno Virus** | | | | | | | | | |
| Author | Biomarker | Study Design | Biopsy Indication | Rejection Type | Sens | Spec | NPV | PPV | AUC |
| Doberer et al (2021)^7^ | TTV | Prospective observational | Surveillance | Acute rejection | 0.91 | 0.24 | 0.15 | 0.94 | 0.64 |
| Doberer et al (2020)^8^ | TTV | Prospective observational | Surveillance and for-cause | Acute rejection and infectious complications | 0.85 | 0.35 | 0.14 | 0.95 | 0.65 |
| Strassl et al (2019)^9^ | TTV | Prospective observational | For-cause |  | 0.94 | 0.27 | 0.19 | 0.96 | 0.67 |

**References)**

1. El Fekih R, Franzen K, Hurley J, et al. An Exosomal mRNA Urine Test for Detection and Risk Stratification of Human Kidney Transplant Rejection. *Kidney Int Rep*. Apr 2025;10(4):1131-1142. doi:10.1016/j.ekir.2025.01.036

2. El Fekih R, Hurley J, Tadigotla V, et al. Discovery and Validation of a Urinary Exosome mRNA Signature for the Diagnosis of Human Kidney Transplant Rejection. *Journal of the American Society of Nephrology : JASN*. Apr 2021;32(4):994-1004. doi:10.1681/asn.2020060850

3. Lim JH, Lee CH, Kim KY, et al. Novel urinary exosomal biomarkers of acute T cell-mediated rejection in kidney transplant recipients: A cross-sectional study. *PloS one*. 2018;13(9):e0204204. doi:10.1371/journal.pone.0204204

4. Seo JW, Lee YH, Tae DH, et al. Development and validation of urinary exosomal microRNA biomarkers for the diagnosis of acute rejection in kidney transplant recipients. *Front Immunol*. 2023;14:1190576. doi:10.3389/fimmu.2023.1190576

5. Matz M, Fabritius K, Lorkowski C, et al. Identification of T Cell-Mediated Vascular Rejection After Kidney Transplantation by the Combined Measurement of 5 Specific MicroRNAs in Blood. *Transplantation*. Apr 2016;100(4):898-907. doi:10.1097/tp.0000000000000873

6. Matz M, Lorkowski C, Fabritius K, et al. Free microRNA levels in plasma distinguish T-cell mediated rejection from stable graft function after kidney transplantation. *Transpl Immunol*. Nov 2016;39:52-59. doi:10.1016/j.trim.2016.09.001

7. Doberer K, Haupenthal F, Nackenhorst M, et al. Torque Teno Virus Load Is Associated With Subclinical Alloreactivity in Kidney Transplant Recipients: A Prospective Observational Trial. *Transplantation*. Sep 1 2021;105(9):2112-2118. doi:10.1097/tp.0000000000003619

8. Doberer K, Schiemann M, Strassl R, et al. Torque teno virus for risk stratification of graft rejection and infection in kidney transplant recipients-A prospective observational trial. *American journal of transplantation : official journal of the American Society of Transplantation and the American Society of Transplant Surgeons*. Aug 2020;20(8):2081-2090. doi:10.1111/ajt.15810

9. Strassl R, Doberer K, Rasoul-Rockenschaub S, et al. Torque Teno Virus for Risk Stratification of Acute Biopsy-Proven Alloreactivity in Kidney Transplant Recipients. *The Journal of infectious diseases*. May 24 2019;219(12):1934-1939. doi:10.1093/infdis/jiz039
